# Local field potentials are induced by visually evoked spiking activity in macaque cortical area MT

**DOI:** 10.1038/s41598-017-17372-4

**Published:** 2017-12-07

**Authors:** Moein Esghaei, Mohammad Reza Daliri, Stefan Treue

**Affiliations:** 1 Cognitive Neurobiology Laboratory, School of Cognitive Sciences, Institute for Research in Fundamental Sciences (IPM), Tehran, Iran; 2 Cognitive Neuroscience Laboratory, German Primate Center - Leibniz Institute for Primate Research, Goettingen, Germany; 3 Neuroscience and Neuroengineering Research Laboratory, Biomedical Engineering Department, School of Electrical Engineering, Iran University of Science and Technology, Tehran, Iran; 4 Faculty of Biology and Psychology, University of Goettingen, Goettingen, Germany; 5 Bernstein Center for Computational Neuroscience, Goettingen, Germany; 6 Leibniz-ScienceCampus Primate Cognition, Goettingen, Germany

## Abstract

Local field potentials (LFP) have been the focus of many recent studies in systems neuroscience. However, the exact neural basis of these signals remains unclear. To address this question, we determined the relationship between LFP signals and another, much better understood, signature of neural activity: action potentials. Specifically, we focused on the relationship between the amplitude of stimulus-induced LFPs and the magnitude of spiking activity in visual cortex of non-human primates. Our trial-by-trial correlation analyses between these two components of extracellular signals in macaque visual cortex show that the spike rate is coupled to the LFP amplitude with a surprisingly long latency, typically 50 ms. Our analysis shows that the neural spike rate is a significant predictor of the LFP amplitude. This limits the functional interpretation of LFP signals beyond that based on spiking activities.

## Introduction

The cerebral cortex encodes sensory information by the activity of neurons, a phenomenon extensively studied using extracellular recording in awake animals. Such recordings can pick up spiking activity, the pattern of action potentials of nearby neurons that encodes information in the rate and temporal distribution of these binary events. For instance, neurons in medial temporal area MT of the macaque visual cortex fire at different rates when stimuli moving at different directions or at different speeds appear in their receptive fields^1^ with most MT neurons preferring a specific direction, speed of motion and binocular disparity^2^. The spiking activity of such sensory neurons is also modulated by high-level cognitive factors such as spatial, feature-based or object-based attention^3–5^. This makes neural spikes a promising target for research on the neural bases of sensory and cognitive functions.

Another component of extracellular signals is local field potentials (LFP) that have attracted attention more recently in neuroscience and neuroengineering studies^6–10^. LFPs are thought to be the sum of synaptic potential fluctuations across thousands of neurons around the tip of a recording electrode^11^ from a volume of up to several hundred cubic micrometers^12–14^. Similar to spiking activity, LFP signals from sensory cortex have been shown to encode stimulus parameters and are also modulated by top-down signals. As an example, recordings from area MT show tuning curves for motion direction in the ‘gamma power’, the power of gamma frequency (40–200 Hz) LFPs^15^. Furthermore, the power of LFPs at this frequency range has been found to increase with switching attention to the receptive field of the recorded site while decreasing at lower frequencies (<20 Hz)^16–18^; However, see^19^ for contrasting results in gamma frequencies. These previous studies suggest that those synaptic activities which create the LFP are not an epiphenomenon of neural spikes, but they influence local modulations of neural processing. Although LFPs and spikes have usually been studied as separate components of extracellular signals, they show similar signatures of sensory parameters.

Spikes and LFPs are highly correlated in many cortical areas^20–23^. This includes correlations between the spike rate and LFP power within a given frequency band as well as the locking of spikes to the LFP phases in different frequency bands. For instance, spike rates of neurons recorded from the rat hippocampus and macaque areas V1 and MT are found to be positively correlated with gamma power across different sensory or cognitive states^15,23–25^; but see^21^ for negative correlations. Similar studies have reported that neural spikes occur mostly at specific phases of LFPs at low (<20 Hz) and high frequencies (30–80 Hz)^19,22,26–29^. Although it has been suggested that spike times could be used to estimate those surrounding synaptic activities that are reflected in the LFP^30^, it remains unclear which of the two components causes the other and correspondingly represents the most direct readout of information transmission.

When spike and LFP components are recorded from the same site, knowing the causality of their interdependence is crucial. This would clarify which of the two signals forms the neural representation earlier. Using information theoretic techniques Besserve *et al*.^31^ suggested that neural spikes and gamma band oscillations in V1 have a causal effect on low frequency LFPs. They also reported causal effects of gamma band on spiking activities when both were recorded from the same site. This study was carried out with anesthetized or passively viewing monkeys. However, for monkeys actively engaged in a behavioral task, the existence of a causality between these signals evoked by a transient stimulus is unclear. Therefore, determining the temporal order of evoked LFP and spikes is of critical relevance.

Here we determined first which of the two signal components (LFP or spiking activity) reaches its peak activity earlier in response to a visual stimulus and second if one of the two predicts the other’s trial-by-trial variability. Recordings were made in the medial temporal area MT of two macaque monkeys while they were performing a visual detection task. We observed that LFP responses reach their maximum activity tens of milliseconds later than spike responses and that a large portion of evoked LFP activity is predicted by the preceding spiking activity.

## Results

**Figure 1:**
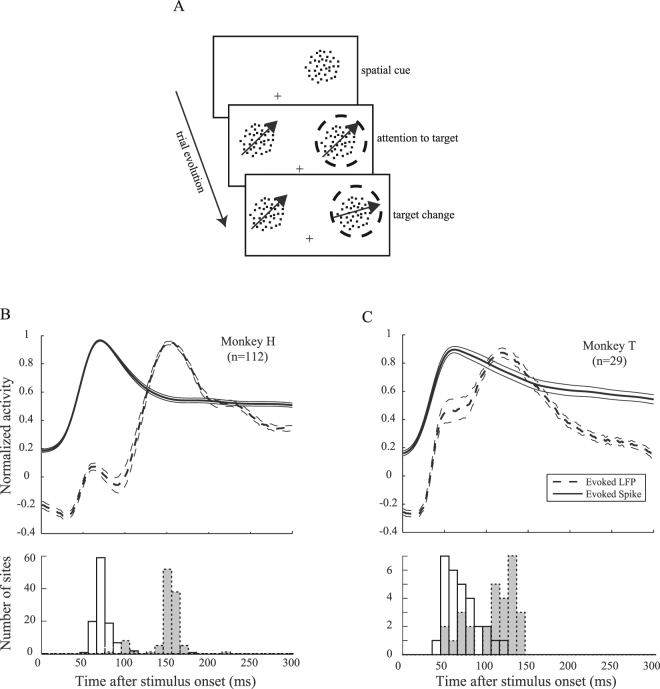
Task design and stimulus-evoked spiking/LFP activity. Panel A shows the behavioral paradigm. The monkeys had to foveate a central fixation point (shown as a cross). Shortly thereafter, an RDP cued the position of the upcoming target stimulus. The monkeys had to detect a subtle change in the color/motion direction of the target RDP (indicated by a dashed circle here) by releasing the lever. Panels B and C present data recorded from each of the monkeys and “n” represents the number of recording sites for each animal. Both spiking activities and LFPs are normalized to the maximum value of each recording site across trials. Standard errors reflect standard error of the mean (SEM). Note that we inversed all LFP values before normalization in order to present the activity relative to the intracellular space (see Materials & Methods for details).

Two macaque monkeys were trained to detect a small change in the direction or color of one of two moving random dot patterns (RDP). Each trial started when the monkeys touched a lever and maintained their gaze at a central point; afterwards a cue was presented showing the position of one of the two upcoming stimuli (target). The monkeys received a juice reward if they released the lever as soon as the target stimulus underwent a small change in its color or direction of motion (Fig. 1A). Details of the paradigm are described elsewhere (see^32^ for monkey H and^3^ for monkey T).

**Figure 2:**
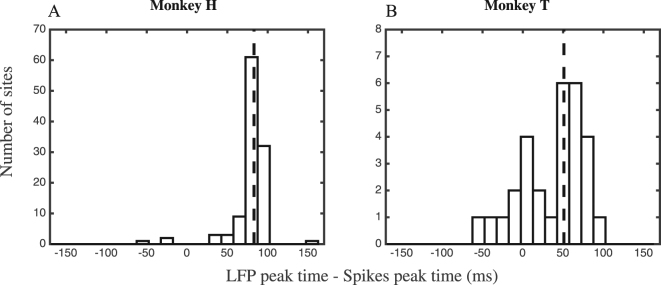
Spiking activity and LFP peak time relative to each other. (**A**,**B**) Each panel shows the histogram of latencies between spiking activity and LFP peaks for one monkey across recording sites. The dashed lines represent the median latencies; which are positive for both monkeys.

We recorded neural activity in the form of spiking activity and LFPs from area MT while one of the two stimuli was presented inside the receptive field (RF) of the neuron being recorded. Figure 1 (upper plots in B and C) shows the normalized spiking activity and LFPs (in inversed values, see Materials and Methods) from monkeys H and T for a 300 ms window after the onset of stimuli. Spike trains are first smoothed and next normalized by the maximum value across trials of each recording site (see Materials & Methods for details). Figure 1 (lower plots in B and C) shows the histogram of times with the largest absolute neural activity. Among a large majority of the sites, the maximum absolute LFP coincides with the peak of the LFP (92% in monkey H, 100% in monkey T, p ≪ 0.001 for both monkeys, sign test), suggesting a largely monotonous profile in LFPs of our dataset. For monkey H the LFP peak occurs at 153 ± 18 (SD) ms while the spiking activity peak is at 74 ± 14 (SD) ms after stimulus onset when calculated for recording sites separately. Similarly, for monkey T the peaks occur at 107 ± 28 ms and 71±20 ms for LFP and spikes, respectively. To determine if there is a systematic difference between the peak times of the evoked LFP and evoked spiking activity, we considered the distribution of differences between the spiking activity and LFP peak times across recording sites. Figure 2A,B shows the histogram of LFP peak time subtracted by spiking peak time for the two monkeys. The dashed line in each panel indicates the median peak time difference for each monkey (83 ms and 51 ms for monkeys H and T, respectively). In both animals the peak of the LFP activity evoked by the stimulus occurs significantly (p < 0.001, signtest) later than the peak of evoked spiking activity. For visually evoked signals, a similar time lag (50 ms) of LFPs relative to the peak spike rate has been observed in primate FEF^33^. Again, with visual stimulation, Tan *et al*. showed a loss of coincidence for deflections of neural membrane potential and LFPs^34^. However, it is unclear whether the spike rate influences LFP amplitude across the substantial delay we observed.

**Figure 3:**
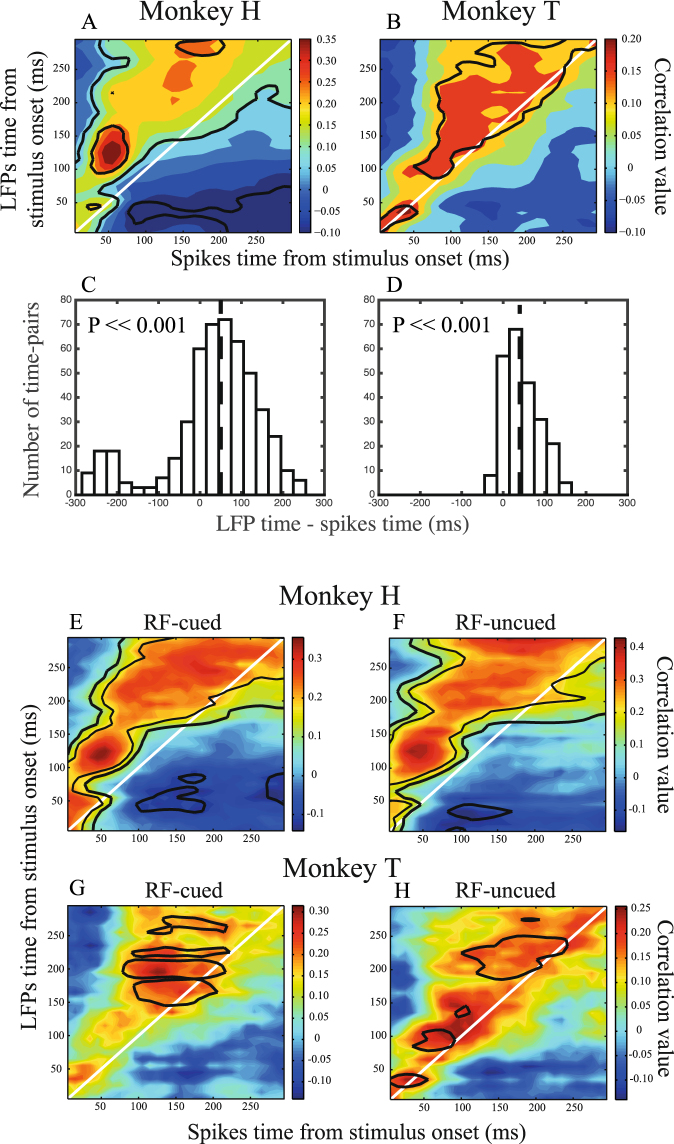
Correlation map across spiking activity & LFP time bins. (**A**–**D**) The top panels (A,B) represent the correlation maps for the two monkeys. X and Y-axes represent the center of the 10 ms time bins (aligned to stimulus onset) for spiking activity and LFP averaging, respectively. For each pair of time bins, colors show the average Pearson correlation of spiking activity and LFP calculated per site across trials with the preferred stimulus. Black borders indicate regions that have statistically significant correlations across recording sites (thicker borders correspond to p < 0.01 (t-test, corrected for multiple comparisons)). White lines connect pairs of time bins with the same X and Y indices and are drawn for illustration. Panels (C,D) present the histogram of “LFP time index – Spikes time index” for the time pairs with significant correlations for each monkey. The dashed vertical lines correspond to the median of each histogram (+40 ms for both monkeys, p ≪ 0.001, sign test). (**E**–**H**) Correlation maps for different cueing conditions. (**E**,**G**) Similar to maps in panels (A,B) but for trials in which the RF location was cued and therefore the stimulus inside the RF (moving in the preferred direction) was the target. (**F**,**H**) Correlation maps for trials where the cue was contralateral to the RF, i.e. the stimulus inside the RF was the distractor. Top and bottom panels correspond to monkeys H and T, respectively. The maps follow the same convention of coding as in panels A & B. Note that correlation values here and in the following figures are computed from the inversed LFP values (see Materials & Methods for details).

In order to investigate a potential influence of the evoked spiking activity on the evoked LFP, we asked whether there is a correlation between the values of the evoked spiking activity and evoked LFP across the task trials. To determine if the cross-time correlation occurs only from spiking activity to LFP, we calculated the inter-trial correlation between spiking activities and LFPs at each site, with the spiking activity and LFP coming from different time bins. This analysis was applied to trials where the preferred stimulus of the recorded neuron was presented. Figure 3A,B shows the correlation values across different pairs of time bins averaged across recording sites. The axes show centers of 10 ms bins stepped by 10 ms starting from stimulus onset. X and Y-axes indicate the time bins from which the mean spiking activity and the mean LFP activity are extracted, respectively. The color code represents the average correlation between mean spiking and LFP activity in a given pair of time bins across trials of a given recording site. Solid black lines characterize time pairs of the map with the mean spike-LFP correlation significantly different from zero across recording sites (Supplementary Figure 1 shows the proportion of significantly correlated sites at each time-pair). Two diagonal white lines connect pairs of time bins with the same indices; so that the area above the lines correspond to time pairs with their LFP index larger than the spike index, conversely for the time pairs below the diagonal lines, the LFP index is smaller than the spike index. Correlation maps of both monkeys show that the majority of time-pairs with a significant correlation occur above the diagonal line indicating that the time index of LFPs follows that of spiking activities in correlated time pairs. To test this directly, we plotted the histogram of differences between the LFP and spike indices for those time pairs with a significant mean correlation (Fig. 3C,D). The X-axes in these histograms represent the LFP time subtracted by the spike time and the dashed vertical lines show the median of this difference across time pairs with significant correlations. Consistent with the rightward shift of the dashed line, both monkeys show a significant difference between the spike and LFP time indices of those time pairs with significant cross-time correlation. In these time pairs, the LFP index follows the spike index by 40 ms for both monkeys (p ≪ 0.001, sign test). This indicates that spiking activity is a significant predictor of future LFP activity.

The unidirectional correlation between spikes and upcoming LFPs could be a side-effect of the correlation of a neuron’s spiking activity with its own following spiking activity. To validate this, we calculated the partial correlation between spikes and LFPs at different times, removing the correlation component associated with spikes at the same time as LFPs (see Materials & Methods for details). This resulted in similar maps as in Fig. 3A,B suggesting that spikes directly influence future LFPs (Supplementary Figure 2A,B).

Furthermore, the LFPs we observed might reflect volume-conducted voltages from other brain areas^35^. To check for this, we focused on monkey H’s data, which contained a sufficient number of simultaneous recordings. We subtracted each electrode’s LFP from that of its neighboring electrode, to remove global effects (Supplementary Figure 3A). Similar to Fig. 3, we calculated the histogram of the difference between LFP and spike time-indices across the time-pairs with a significant correlation (Supplementary Figure 3B). Similar to Fig. 3C,D, the LFP time index was significantly larger that the spike time index (p ≪ 0.001, sign test). This indicates that the unidirectional correlation between spikes and the following LFP is not due to voltages volume-conducted from other brain areas. It may be further argued that the asymmetric pattern of cross-time correlations relative to the diagonal line depends on the behavioral condition of the trials the monkeys were performing. In each trial of the task, either the stimulus inside or outside the RF was cued, and the monkeys were rewarded to report changes in only the cued stimulus. We carried out similar analyses as in Fig. 3A,B on each of the two trial types: RF-cued and RF-uncued, where the RF’s position or the position contralateral to the RF was cued, respectively. Similar to Fig. 3A,B, here we focused on trials where the preferred stimulus of the recorded neuron was presented. Fig. 3E–H shows the resulting maps for each of the cueing conditions for both monkeys. Top and bottom panels present the correlation maps for monkeys H and T, respectively. The left and right panels present the maps for RF-cued and RF-uncued trials, respectively. For both monkeys the asymmetric pattern relative to the diagonal line can be perceived in both cueing conditions similar to when trials of both cueing conditions were pooled (Fig. 3A,B) and a majority of time pairs with significant correlations are above the diagonal line. The LFP time index at these time pairs is significantly greater than the spike time index for the RF-cued and RF-uncued conditions (50 ms for each animal, p ≪ 0.001, sign test). This result suggests that the asymmetry of the correlation map relative to the diagonal line is not due to the monkey’s cognitive state.

**Figure 4:**
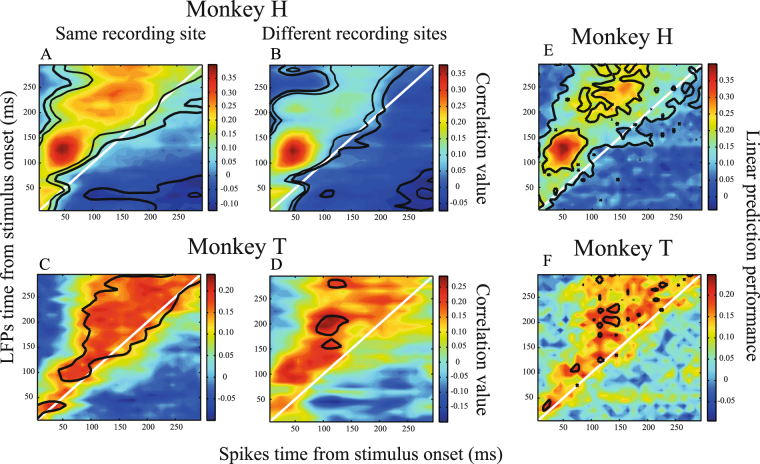
Correlation maps for spiking activity/LFP from same/different recording sites and linear model prediction of LFPs. (**A**,**C**) Similar correlation maps as in Fig. 3A,B where spiking activity and LFP come from the same recording site. (**B**,**D**) Correlation maps based on spiking activity and LFP recorded from different MT sites simultaneously. Top and bottom panels correspond to monkeys H and T, respectively. (**E**,**F**) Linear model predictions of LFPs based on spiking activity. Performance of estimating LFPs based on spiking activity for different pairs of intervals relative to stimulus onset is shown. Colors code the correlation of a linear estimator’s output with original LFPs at each spike-LFP time pair, and each panel reflects the results for one monkey. The diagonal white line in each panel indicates simultaneous spike-LFP pairs. Black borders specify time pairs with statistically significant estimation performances across recording sites, quantified as the correlation between the estimated and original LFP amplitudes (p < 0.05, t-test, corrected for multiple comparisons).

In order to ensure that our result is not due to spectral leakage of the spike waveforms into LFPs of the same electrode, the same analysis as in Fig. 3A,B was applied to LFP and spiking activities recorded from separate electrodes simultaneously. Figure 4B,D illustrates the correlation maps for LFP and spiking activity from a different electrode for monkey H (Fig. 4B) and monkey T (Fig. 4D). Compared to the correlation maps in Fig. 3A,B (shown also in Fig. 4A,C) (corresponding to the condition that LFP and spiking activity were recorded from the same site) the asymmetry relative to the diagonal line is preserved. Consequently, the LFP time index at those time pairs with significant correlations is larger than the spiking activity time index (40 ms for both monk+eys, p ≪ 0.001 for both monkeys, sign test). This suggests that the correlation between spiking activity and following LFP activity is not due to common spectral components of spiking activity and LFP signals when recorded from the same site.

Given the high trial-by-trial correlation between spiking activity and the following LFP amplitude, we next hypothesized that earlier spiking activity should predict following LFP amplitudes. Therefore, we randomly selected half of the trials for each site and fitted a linear model on the spike-LFP pairs coming from different time slots. Next the linear model was used to estimate LFP amplitude based on spiking activity in the remaining trials. The correlation of the estimated LFP and the original LFP amplitudes was calculated as a measure of the linear estimator’s prediction performance (see Materials & Methods for details). Results are shown in Fig. 4E,F, where each panel presents the prediction performance of the linear estimation for one monkey and color codes the performance of the linear estimation at each spike-LFP time pair. The maps show a similar pattern as in Fig. 3A,B with asymmetry relative to the diagonal line and a significantly higher LFP time index than spiking activity time index for time pairs with a significant prediction performance (p ≪ 0.001, sign test). This shows a uni-directional functional link between spiking activity and LFP amplitudes occurring with a substantial delay.

**Figure 5:**
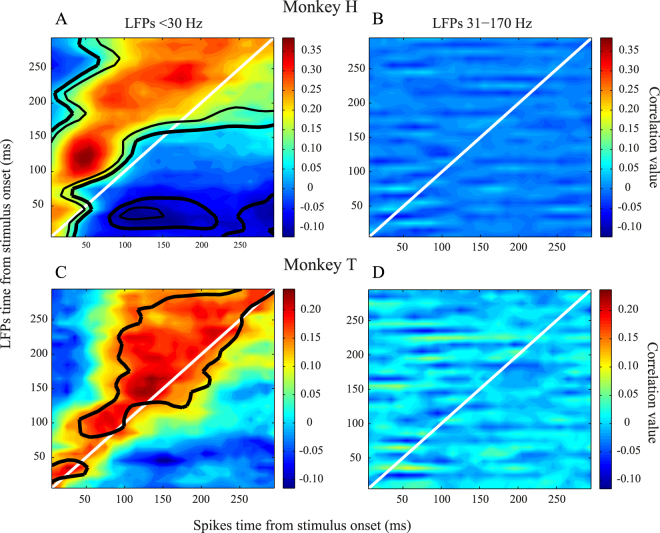
Correlation maps based on LFPs of different frequency bands. (**A**,**C**) LFPs of frequencies lower than 30 Hz were used to create the correlation maps. (**B**,**D**) High frequency LFPs with frequencies higher than 30 Hz were considered here. Top and bottom panels correspond to monkeys H and T, respectively. Maps follow the same convention of color coding as in Fig. 3A,B.

So far we considered LFP across all frequencies. Next we asked whether the correlation between spiking activity in a given time and LFP in following intervals occurs at any specific frequency band of LFP in particular. Therefore, we divided LFP into frequencies higher than 30 Hz and frequencies lower than 30 Hz. This division addresses the distinction between the functional role of these two frequency bands in neural mechanisms of visual processing and attention in area MT^18,32^. Figure 5 presents the spike-LFP correlation maps for each of these two frequency bands; the left and right panels focus on frequencies lower and higher than 30 Hz, respectively and figures in each row show the maps for one monkey. As shown in the right panel, there is no statistically significant correlation between spiking activity and LFP at frequencies higher than 30 Hz among any of the pairs of time intervals we have studied. In contrast as shown in the left panel, the properties of correlation maps remain similar to the original maps calculated based on the full frequency spectrum of LFP (Fig. 3A,B) in terms of asymmetry relative to the diagonal line, and for both monkeys the time pairs with a significant cross time correlation rest above the diagonal line (p ≪ 0.001, sign test). This suggests that the cross-time correlation of spiking activity and LFP magnitude holds only for frequencies less than 30 Hz that are linked to shared neural activities across larger volumes of cortex compared to that of frequencies higher than 30 Hz.

**Figure 6:**
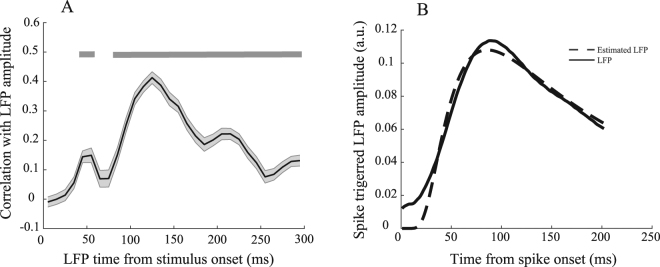
Spike-LFP link in time. (**A**) Correlation of spikes at 55 ms with surrounding LFPs. X-axis shows the times that LFPs come from and Y-axis presents the mean correlation value of spikes at a 10 ms time window around 55 ms with the LFPs. Those times with a significant correlation across recording sites are indicated by a grey bar (corrected for multiple comparisons). Error bars show SEM. (**B**) Spike-triggered LFP for spikes between 0–100 ms after stimulus onset. To show how spikes rule the upcoming LFP, we have averaged the z-scored LFPs following spikes across the recorded sites from both monkeys (solid line). The average spike-triggered LFP is estimated using a Weibull function (shown in dashed line).

We next asked how spikes affect LFPs occurring tens of milliseconds after the spikes. For this, we focused on the data of monkey H, because of the availability of a larger number of sites, and on those spikes with the highest spike-LFP correlation (spike index = 55 ms, Fig. 3A). Figure 6A plots the correlation of those spikes with the LFPs. This curve shows the highest spike-LFP correlation for LFPs 125 ms after the stimulus onset. It should be noted that there is an earlier peak in the correlation curve at about 55 ms which reflects the instantaneous interaction between post-synaptic potentials and spikes (confirming the significantly correlated time-pairs on the diagonal line (Fig. 3A,B)). This instantaneous interaction provides an explanation as to why the onset of stimulus evoked LFP and spiking activity occur simultaneously after stimulus presentation (Fig. 1B,C). Furthermore, the correlation shows an oscillatory pattern (<20 Hz) in time, suggesting that not only spikes influence the LFP at different upcoming times in a non-uniform manner, but that this influence is dependent on the phase of an oscillatory state, phase-locked to the stimulus onset. Further study needs to characterize the potential link of this oscillatory state with low frequency LFPs. Finally, it should be possible to extract a filter allowing for the prediction of LFPs from spikes. We estimated this kernel by estimating the spike-triggered LFP for spikes between 0–100 ms following the stimulus onset (the interval with the maximum MU-LFP correlation) using a Weibull function (Fig. 6B):

$$y=c\ast {(\frac{x}{a})}^{b-1}\ast \exp (-{(\frac{x}{a})}^{b})$$

where x is time (ms) and y is the estimated spike-triggered average LFP. We found a, b, and c to be 182, −0.9, and 0.19, respectively. Consistent with a study^36^ in rodents, this suggests that the LFPs following spikes can be predicted, using a convolution-based method.

## Discussion

LFP signals and their possible causal link to cognitive aspects of brain functions have attracted wide interest. However, the exact origin of these signals and their functional role, especially in sensory areas remains unclear. Here we investigated the relationship between action potentials and LFPs in area MT of macaque visual cortex in search for a possible causal link and its directionality. By calculating the trial-by-trial correlation between the two signal components across time bins with different intervals in between, we show that spiking activity predicts LFP activity in the transient part of neural responses. We observed a significant trial-by-trial correlation between the LFP and preceding spiking activity. Using linear estimation, we show that the spiking activity at a given time can predict the upcoming LFP amplitude. This suggests that evoked spiking activity has a significant role in determining LFP activity. This effect is observed across different behavioral conditions and cannot be attributed to spectral contaminations between spiking and LFP activity recorded from the same site, as only low frequency (<30 Hz) LFPs show this link to spiking activity. Assuming that a similar principle of information-representation holds across different sensory areas, we assume that our findings could be extended to similar sensory areas as well.

We interpret our data to show that spiking activity induces LFP unidirectionally. To confirm this, we calculated trial-by-trial correlations across successive time slots. It may be argued that a third factor is causing the trial-by-trial variability in both spiking activities and LFPs. The spiking input coming from upstream visual areas (V1 and V2) could be such a factor, inducing both spiking activity and LFP. However, this input would first influence post-synaptic potentials and only then the spiking activity in area MT. Since post-synaptic potentials constitute the main component of LFPs^11^, the highest cross-time correlation between spiking activity and LFP should occur at time pairs where the spiking activity time follows the LFP time. Our results show that this is clearly not the case, suggesting that spiking activity in area MT rather than an external source of spiking input governs fluctuations in LFP of the area. Nevertheless, to directly study if evoked spiking activity causes the LFP-reflecting synaptic response, one would ideally selectively de-activate single neurons and inspect the effect on the evoked LFP. Although previous studies have shown that selectively activating neurons using optogenetics induces modulation in LFP power^37^, similar studies are necessary to investigate if visually evoked spiking activity is the essential cause of visually evoked synaptic activities reflected by LFPs. Our result is in line with a previous study^38^ that shows, although there is a component of LFP that can predict either the intracellularly recorded depolarization or the action potential, the LFP component following this depolarization has a higher amplitude and lasts longer. It is further in agreement with a report on causality effects of spiking activities on low frequency LFP in macaque V1^31^, especially in the sense that low frequencies are emphasized in transient neural responses (Fig. 1B,C). However, there are differences in the time scale of the effect between this study and our data.

The median delay between spiking activity and LFPs at time bin pairs with a significant correlation is about 40 ms for both monkeys (Fig. 3C,D), i.e. fluctuations in spiking activity take tens of milliseconds to be reflected in the amplitude of LFP. Despite the similarity in the magnitude of the delay, there is a noticeable time-lag between the peaks of the average LFP in the two animals (33 ms, p < 0.0001, permutation test (with 100,000 repetitions); 151 ms and 118 ms for monkeys H and T, respectively). We speculate that this difference reflects differences between the tasks of the two monkeys; for monkey H we showed a full-sized RDP, while for monkey T we showed a small RDP at a small eccentricity close to the fixation point.

Although we found a delay up to 40 ms between spikes and LFP, Besserve *et al*. reported that the largest component of the causality effect has a time lag of only a few milliseconds. Given the influence of wakefulness on the amplitude of evoked activity and its trial by trial variability^39,40^, this difference may be because their recordings were carried out under anesthesia whereas our monkeys were actively engaged in a visual detection task during the recordings. Using an alternative approach Rasch *et al*. showed that neural spikes can be used to linearly estimate LFP^30^. Consistent with our results they found that the estimation is best done in the time scale of up to 200 ms from spike occurrence. However, the kernel they introduce for estimating LFPs produces a negative trial-by-trial correlation between spike rate and the following LFPs (with reversed amplitude). This is in disagreement with the positive correlations in our data (Fig. 3A,B; values above the diagonal line). Again, because their data were recorded while the monkeys were either anesthetized or passively viewing a movie and given the suppressed coding capacity of neurons under non-wakefulness^41^, their result cannot be generalized to when animals actively attend. Furthermore, our observation concerning the long time scale in which spikes influence LFP suggests that neural spikes modulate LFP in time scales much longer than that of synaptic transmission. One might speculate that the incoming sensory spikes cause a short-term network-level modification in MT that influences LFP-generation on a time scale of tens of milliseconds. Based on the previous observation of differed LFP profiles across cortical layers^42^, it could be argued that the unidirectional induction of LFP by spiking activity is layer-specific. While we do not know the cortical layer of our recordings, the dominance of sites with a larger evoked peak rather than evoked trough shows that the LFPs are homogeneous across recording sites (Fig. 1B,C). Nevertheless, this possibility remains that our LFPs are recorded only from distal dendritic regions, inducing the time lag with spikes. Although this is unlikely given the high number of recordings in our dataset, future recordings from separate cortical layers of area MT could give a clear view on how the activity of neurons in each layer contributes to the LFP.

To summarize: since the biophysical and neural bases as well as the functional correlates of LFPs are not fully understood, we investigated the interaction between these signals and action potentials, the much better understood signature of neural activity and coding. Our results show a strong unidirectional influence of spiking activity on LFPs during transient neural responses, indicating that a considerable component of LFP is explained by spiking activity. This suggests that LFPs, as well as other types of field potentials (ECoG, EEG, …) are an epiphenomenon, i.e. an indirect, rather than direct measure of brain states.

## Materials and Methods

### Animal welfare

Research with non-human primates represents a small but indispensable component of neuroscience research. The scientists in this study are aware and are committed to the great responsibility they have in ensuring the best possible science with the least possible harm to the animals^43^. All animal procedures and methods of this study have been approved by the responsible regional government office (Niedersaechsisches Landesamt fuer Verbraucherschutz und Lebensmittelsicherheit (LAVES)) under the permit numbers 33.42502/08-07.02 and 33.14.42502-04-064/07 and were carried out in accordance with all applicable laws and regulations. The animals were group-housed with other macaque monkeys in facilities of the German Primate Center in Goettingen, Germany in accordance with all applicable German and European regulations. The facility provides the animals with an enriched environment (incl. a multitude of toys and wooden structures^44^), natural as well as artificial light, exceeding the size requirements of the European regulations, including access to outdoor space. Surgeries were performed aseptically under gas anesthesia using standard techniques, including appropriate peri-surgical analgesia and monitoring to minimize potential suffering.

The German Primate Center has several staff veterinarians that regularly monitor and examine the animals and consult on procedures. During the study the animals had unrestricted access to food and fluid, except on the days where data were collected or the animals were trained on the behavioral paradigm. On these days the animals were allowed unlimited access to fluid through their performance in the behavioral paradigm. Here the animals received fluid rewards for every correctly performed trial. Throughout the study the animals’ psychological and veterinary welfare was monitored by the veterinarians, the animal facility staff and the lab’s scientists, all specialized in working with non-human primates. The two animals were healthy at the conclusion of our study and were subsequently used in other studies.

### Behavioral task and recording

Two male macaque monkeys were trained to fixate a central fixation point and touch a lever to start each trial. Eye movements were monitored using a high-speed video-based eye tracker at a sampling rate of 230 Hz (ET49, Thomas Recording, Giessen, Germany). Each trial started with presenting a cue on the screen indicating one of upcoming moving random dot patterns (RDP) as the relevant stimulus (target). For monkey H, the cue was a static RDP shown at the same position and with the same size as the target and for monkey T it was a small RDP close to the fixation point and on an imaginary line connecting the fixation point to the upcoming target stimulus. After the cue was removed, two moving RDPs were presented at equal eccentricities in opposite visual hemifields and the monkeys had to detect a small direction (monkey H) or color/direction change (monkey T) in the target RDP. This change could happen at a random time, not earlier than 500 ms after stimulus onset and the monkeys were rewarded for releasing the lever in a time window between 100–650 ms after the target change. If the animal’s gaze deviated from the fixation point within a trial, the trial would terminate without a reward. For more details about the behavioral paradigm and the behavioral performance for monkey H see^32^ and for monkey T see^3^.

While the monkeys performed the task, we recorded multi-unit spiking activities and local field potentials (LFP) from area MT using a five-channel multi-electrode recording system (Mini-Matrix, Thomas Recording, and Plexon multi-channel acquisition system (MAP), Plexon Inc.). The electrode signal was split into LFP and spike components by hardware filters. The LFPs were amplified and digitized at 1 kHz, while spikes were amplified and digitized at 40 kHz. Multi-unit spikes were determined by voltage thresholding. We recorded from up to all five electrodes (with the impedance of 2 MΩ arranged linearly separated by 305 *μ*m) simultaneously. In sessions with simultaneous recordings, we made sure that the RFs of the different multi-units overlap sufficiently for all to contain the stimulus placed in the RF. Recording sites were determined to be located in MT by their position in the cortex, receptive field location and size, as well as the neurons’ tuning for linear motion directions. For monkey H, the RDPs could move in one of 8 equally spaced directions between 0° and 360° and for monkey T, the motion direction would be either the preferred or anti-preferred direction of the neuron under study.

### Data analysis

All analyses were carried out in MATLAB (Mathworks, Natick, MA). To generate smoothed spiking activities (Fig. 1B,C), a Gaussian function (σ = 15 ms) was convolved with the spike trains and the outcome was normalized per site to the maximum value across trials. Other Gaussian functions (σ = 5, 10) did not alter our main results (Fig. 3A–D). To avoid any phase lag enforced by the recording headstage, we aligned the phases of LFP signals by applying a time-reversed filter on the LFP, which was built upon measurements of the recording hardware’s phase shift values (provided by Plexon, Inc: FPAlign utility guide-version 1.0)^45^. The 50 Hz line noise, the 76 Hz noise due to the monitor refresh rate and its periodical (152 Hz) were extracted and removed from the original signal using MATLAB’s *idealfilter* routine (a non-causal ideal bandpass filter), which extracts a given frequency component after applying Fourier transform on the signal and next applying an inverse-Fourier transform over it. We controlled for multiple comparisons using Bonferroni correction. LFPs were first inversed for all trials and next normalized by the maximum value across trials separately for each recording site. All our figures show these inversed values of the LFPs in order to present the activity relative to the intracellular space. To study the spike-LFP link, we calculated the cross-time spike rate-LFP trial-by-trial correlation. This measure tolerates any non-stationarity enforced by the onset of stimulus (in contrast to approaches such as spike-triggered LFP). In the correlation maps we identified time pairs with a significant correlation by testing for each time pair, if the correlation values across sites were significantly different from zero using a sign test. Partial correlation between spiking activity and LFP of a time pair was calculated by measuring the correlation between the two residual values resulting from a) the linear regression of spiking activity with the spiking activity simultaneous to LFP and b) the linear regression of LFP with the spiking activity simultaneous to it. For calculating correlation maps based on different LFP frequencies (Fig. 5), we first subtracted the average evoked LFP from each trial’s LFP for every site. This ensures that filtering LFPs into different frequencies is not contaminated by the transient response evoked with stimulus onset. Signals were filtered using MATLAB’s *idealfilter* routine. Similar results were achieved using a zero-phase FIR filter (eegfilt function^46^; with the filter order of 3*(sampling_rate/low_cutoff_freq) and assuming each given LFP signal as one epoc). For the linear estimation of LFPs based on spiking activity (Fig. 4E,F) for each site at each spike-LFP time pair, first, the trials were divided randomly into two equal-sized groups, second we estimated a linear model using MATLAB *fitlm* function based on one of the groups and finally predicted the LFP of the second group based on its spiking activity data using the model computed from the first group (with the aid of MATLAB *predict* function). In order to assess the performance of this prediction method, the Pearson correlation between the predicted values and the original LFP data was calculated.

### Data availability statement

The datasets generated during the current study are available from the corresponding author on reasonable request.

## Electronic supplementary material

Supplementary figures
