# Supplementary material for: Local field potentials are induced by visually evoked spiking activity in macaque cortical area MT
Source: Sci Rep. 2017 Dec 7;7:17110. doi: 10.1038/s41598-017-17372-4 (PMC5719360; doi:10.1038/s41598-017-17372-4)
Supplement: Supplementary file 1 — Supplementary figures [file 41598_2017_17372_MOESM1_ESM.pdf]

Supplementary figures

Local field potentials are induced by visually evoked spiking activity in macaque cortical area MT

Moein Esghaei, Mohammad Reza Daliri, Stefan Treue

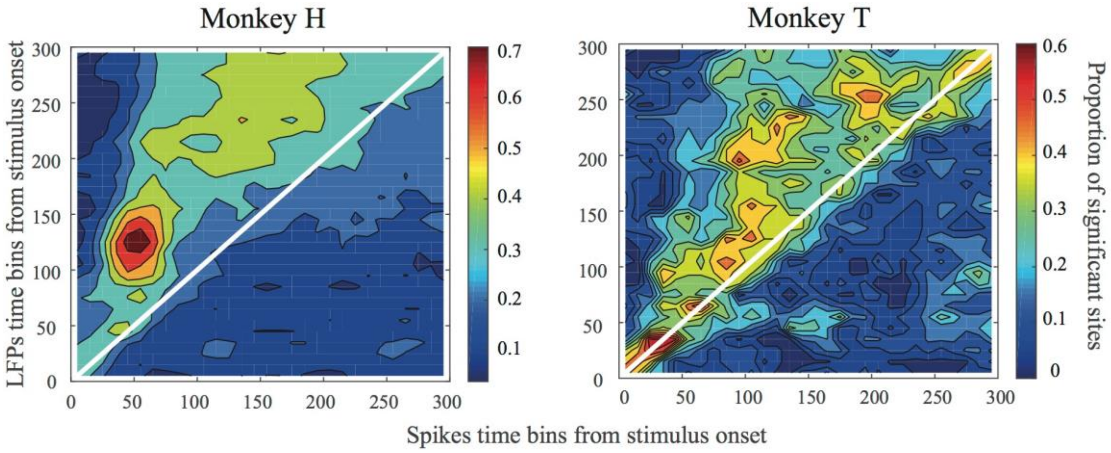

Supplementary figure 1: Proportion of significantly correlated sites. Each map presents for each monkey, the proportion of sites with a significant correlation ($p < 0.05$) between spiking activity and LFP at each time-pair.

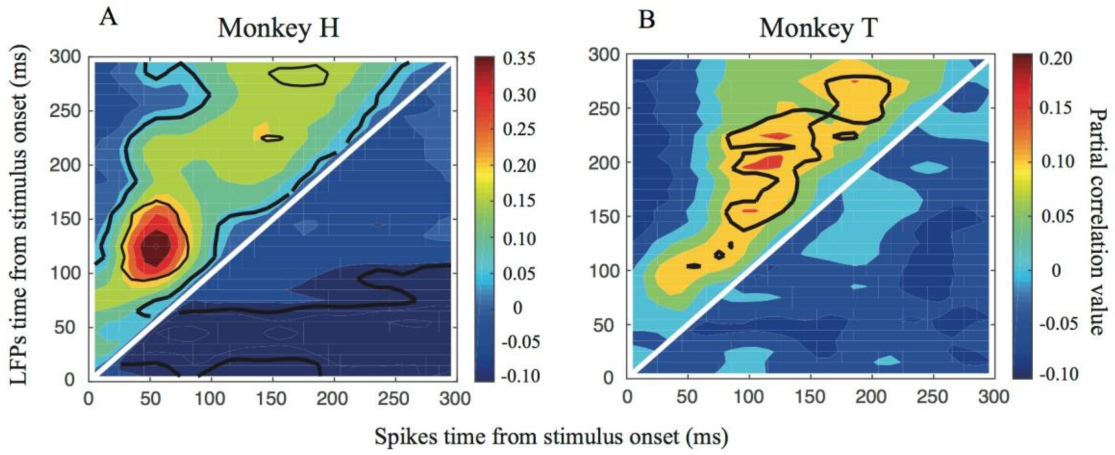

Supplementary figure 2: Partial correlation maps. (A, B) Partial correlation is calculated between spiking activity and LFP at different time pairs, controlling for the indirect role of spikes at the same time as LFPs on the correlation of spikes on future LFPs. Each panel shows the partial correlation map for one monkey. Solid borders indicate time pairs with significant partial correlations.

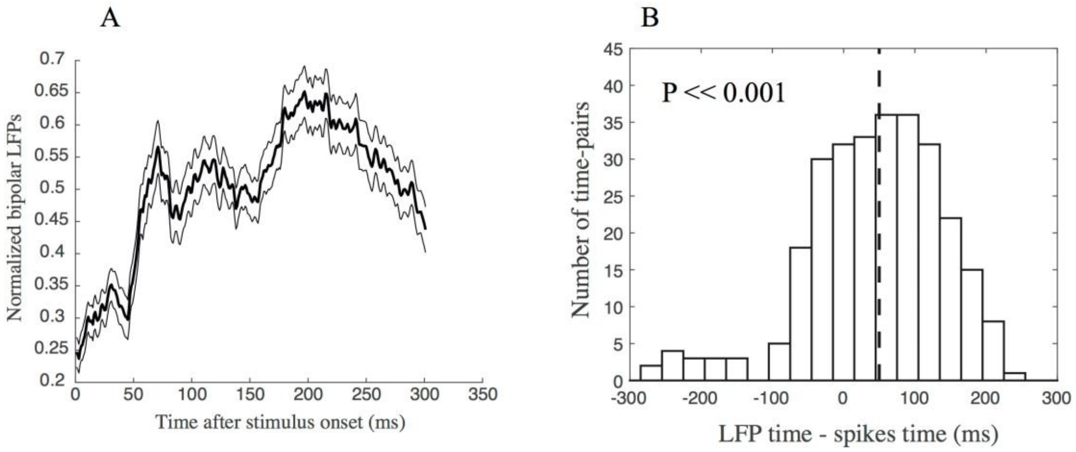

Supplementary figure 3: Control for volume-conduction. (A) LFP variations limited to the local vicinity of each recording site, calculated by subtracting the LFPs from neighboring recording electrodes. Here we subtracted each electrode's LFP magnitude at a time from that of the closest neighboring electrode with the same impedance property (see details on the electrodes in Materials and Methods) (representing focal LFPs). This analysis was performed on monkey H's data since we had enough number of parallel recordings in each session, only for this monkey (53 electrode pairs). The Y-axis represents the absolute difference between the neighboring electrodes' voltages, normalized by the maximum voltage-difference per site. (B) Spike-LFP correlation calculated based on the focal LFPs. We calculated the spike-LFP correlation map (following similar steps as for figure 3A) and plotted the histogram of differences between LFP-spike time-indices in significantly correlated time-pairs (similar to the analysis for figure 3C). We came up with consistent results as in figure 3C & D, with the LFP index being significantly larger than the spike index in time-pairs with a significant correlation (median = 50 ms, $p << 0.001$ sign test). This suggests that our finding on the time lag between spike and LFP is not associated to the volume-conductance.
